# Upstream molecular signaling pathways of p27(Kip1) expression in human breast cancer cells *in vitro*: differential effects of 4-hydroxytamoxifen and deficiency of either D-(+)-glucose or L-leucine

**DOI:** 10.1186/1475-2867-11-31

**Published:** 2011-09-09

**Authors:** Isao Eto

**Affiliations:** 1 Department of Nutrition Sciences, University of Alabama at Birmingham, Birmingham, Alabama, USA

## Abstract

**Background:**

The objective of this study was to investigate whether the levels of glucose or certain amino acids could regulate the expression of a cell cycle repressor protein p27(Kip1), thereby dictating the risk of cancer in either obesity or caloric/dietary restriction. Previously, we identified and reported four different upstream molecular signaling pathways of p27 expression in human breast cancer cells. We called these four pathways as pathway #1, #2, #3 and #4. We found that 4-hydroxytamoxifen - but not tamoxifen - up-regulated the expression of p27 using pathway #1 which consisted mainly of receptor tyrosine kinases and mTORC1. We now investigate, using 4-hydroxytamoxifen as a reference anti-cancer agents, whether (a) the moderate increase in the concentration of D-(+)-glucose could down-regulate and, conversely, (b) the deficiency of D-(+)-glucose or certain L-amino acids could up-regulate the expression of p27 in these cells using pathway #2 which consists mainly of AMPK and mTORC1.

**Results:**

Using human MDA-MB-231 breast cancer cells *in vitro*, these hypotheses were tested experimentally by performing p27-luciferase reporter transfection assays and western immunoblot analyses. The results obtained are consistent with these hypotheses. Furthermore, the results indicated that, although 4-hydroxytamoxifen used primarily pathway #1 to down-regulate the phosphorylation of 4E-BP1 and up-regulate the expression of p27, it also secondarily down-regulated the phosphorylation of S6K1. In contrast, the deficiency of D-(+)-glucose or L-leucine used primarily pathway #2 to down-regulate the phosphorylation of S6K1, but they also secondarily down-regulated the phosphorylation of 4E-BP1 and up-regulated the expression of p27. Finally, deficiency of D-(+)-glucose or L-leucine - but not 4-hydroxytamoxifen - up-regulated the expression of mitochondrial ATP5A and SIRT3.

**Conclusions:**

(a) 4-Hydroxitamoxifen used primarily pathway #1 to up-regulate the expression of p27. (b) Moderate increase in the concentration of D-(+)-glucose used primarily pathway #2 to down-regulate the expression of p27. (c) Deficiency of D-(+)-glucose or L-leucine also used primarily pathway #2 to up-regulate the expression of p27. (d) Deficiency of D-(+)-glucose or L-leucine - but not 4-hydroxytamoxifen - up-regulated the expression of mitochondrial ATP5A in the Complex V of respiratory oxidation-phosphorylation chain and mitochondrial SIRT3. The SIRT3 is one of the seven mammalian anti-aging as well as anti-metabolic sirtuins.

## Background

The risk of developing cancer is increased in obesity where the serum levels of glucose, certain amino acids, insulin and other growth factors tend to be elevated. Conversely, the risk of developing cancer is decreased in caloric/dietary restriction where the serum levels of these metabolites tend to be reduced. The objective of this study was to investigate whether the levels of glucose or certain amino acids could regulate the expression of a cell cycle repressor protein p27(Kip1), thereby dictating the risk of cancer in either obesity or caloric/dietary restriction.

p27 is a member of the family of cyclin-dependent kinase (CDK) inhibitors (CDIs). p27 binds to certain cyclin/CDK complexes, arrests the cell cycle progression from G1 to S phase and inhibit DNA replication. It is known that a relatively large number of nutritional and chemopreventive anti-cancer agents - including 4-hydroxytamoxifen - specifically up-regulate the expression of p27 in both estrogen receptor (ER)-positive and -negative human breast cancer cells *in vitro *[1,2]. It is also known that some other anti-cancer agents specifically up-regulate the expression of p27 in either ER-positive or -negative human breast cancer cells *in vitro *[1,2].

p27 exhibits a set of unique characteristics that are not seen in other G1-to-S phase cell cycle regulatory proteins [1,2]. First, various anti-cancer agents specifically up-regulate the expression of p27 without directly affecting expression of other G1-to-S phase cell cycle regulatory proteins including INK4s, p57(Kip2), p21(Cip1Waf1), D-type cyclins, cyclin E, cyclin A, CDK2, CDK4 and CDK6 [1-3]. Secondly, the degree of up-regulation of the expression of p27 in human breast cancer cell lines *in vitro *by these anti-cancer agents linearly and positively correlates with the degree of inhibition of methylnitrosourea (MNU)-induced rat mammary adenocarcinoma *in vivo *by the same anti-cancer agents [2]. This linear and positive correlation could not be held if a particular anti-cancer agent must be converted to an active metabolite *in vivo *in order to up-regulate the expression of p27. An example of such anti-cancer agent is tamoxifen which must be converted to 4-hydroxytamoxifen *in vivo *to specifically up-regulate the expression of p27. Lastly, unlike other G1-to-S phase cell cycle regulatory proteins, expression of p27 is not regulated at the level of transcription, but primarily at the level of translation. It was observed in the 1980s and 1990s that, during the progression of cell cycle, the level of p27 protein expression oscillated cyclically, but the level of p27 mRNA remained constant. This observation led investigators to suggest that, during the cell cycle, expression of p27 is regulated primarily at the level of translation [4-10]. It was also proposed that the expression of p27 during the progression of cell cycle could be regulated by various other post-translational mechanisms including ubiquitin-proteasome-induced degradation [11-14], complex formation [15], subcellular localization [16-21] and phosphorylation [21-23]. Based on the results of our previous studies [1,2], we believe that a large number of anti-cancer agents up-regulate the expression of p27 primarily by activating the rate of translation initiation of p27 mRNA.

Despite all these information, however, very little is known about the upstream molecular signaling pathways of how various anti-cancer agents specifically up-regulate the expression of p27 in human breast cancer cells *in vitro*. Previously, we identified and reported four different upstream molecular signaling pathways of p27 expression by using p27-luciferase reporter plasmids, western immunoblot analysis and numerous specific inhibitors and stimulators of p27 expression [1,2]. (We will call these four pathways as #1, #2, #3 and #4.).

We also reported previously that, in both ER-positive and -negative human breast cancer cells *in vitro*, 4-hydroxytamoxifen (4-OH-tamoxifen) - but not tamoxifen - up-regulated the expression of p27 by using pathway #1 which consists mainly of receptor tyrosine kinases (RTKs) and mammalian target of rapamycin complex 1 (mTORC1) [2].

We now hypothesize that moderate increase in the concentration of D-(+)-glucose down-regulates the expression of p27 in human breast cancer cells *in vitro *by using pathway #2 which consists mainly of 5'-AMP-activated protein kinase (AMPK) and mTORC1. Conversely, we also hypothesize that deficiency of D-(+)-glucose or certain L-amino acids up-regulates the expression of p27 in these cells by using the pathway #2

To test these hypotheses, we performed p27-luciferase reporter transfection assays and western immunoblot analyses using ER and LKB1-double negative human MDA-MB-231 breast cancer cell *in vitro*. The results obtained were consistent with these hypotheses. Additional results were also obtained that indicated that deficiency of D-(+)-glucose or L-leucine - but not 4-hydroxytamoxifen - up-regulated the expression of mitochondrial ATP Synthase α chain (ATP5A) in the Complex V of respiratory oxidation-phosphorylation chain and mitochondrial SIRT3 in these cells. The SIRT3 is one of the seven mammalian anti-aging and anti-metabolic sirtuins.

## Results

### 4-Hydroxytamoxifen - but not tamoxifen - up-regulated the expression of p27 in estrogen receptor (ER) and LKB1-double negative human MDA-MB-231 breast cancer cells in vitro

**Figure 1:**
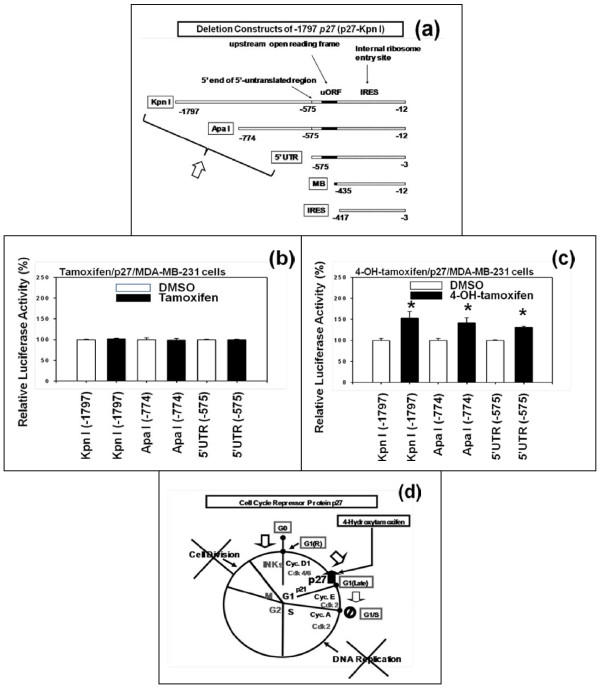
**4-Hydroxytamoxifen - but not tamoxifen - up-regulated the expression of p27 in estrogen receptor (ER) and LKB1-double negative human MDA-MB-231 breast cancer cells in vitro**. (a) Schematic drawing of the luciferase reporter plasmids containing the following proximal 5'-upstream regions of the *p27 *gene, namely -1797 p27 (p27-Kpn I), -774 *p27 *(p27-Apa I), and -575 *p27 *(p27-5'UTR). (b) Tamoxifen did not up-regulate the relative luciferase activity of p27-Kpn 1, Apa I or 5'UTR in MDA-MB-231 cells (c) 4-Hydroxytamoxifen up-regulated the relative luciferase activity of the three p27- luciferase reporter plasmids in MDA-MB-231 cells. In all experiments, the cells were exposed to either DMSO or 1 μM each of tamoxifen or 4-hydroxytamoxifen for 24 hours. All assays were performed in triplicates and repeated three times. (d) Schematic diagram showing how 4-hydroxytamoxifen could up-regulate the expression of p27, arrest the progression of cell cycle from G1 to S phase, and inhibit DNA replication.

The effects of 4-hydroxytamoxifen (4-OH-tamoxifen) and tamoxifen on the expression of p27 in human breast cancer cells *in vitro *were investigated using p27-luciferase reporter plasmids containing the following proximal 5'-upstream regions of *p27 *gene, namely -1797 *p27 *(p27-Kpn I), -774 *p27 *(p27-Apa I), and -575 *p27 *(p27-5'UTR) (Figure 1a). These plasmids were transfected into ER and LKB1-double negative human MDA-MB-231 breast cancer cells *in vitro *and then the transfected cells were exposed to DMSO or 1 μM each of tamoxifen or 4-OH-tamoxifen for 24 hours. The results indicated that tamoxifen did not up-regulate the relative luciferase activity of p27 (Figure 1b), but 4-OH-tamoxifen up-regulated it in these cells (Figure 1c). Previously, we reported essentially the same results using ER and LKB1-double positive human MCF7 breast cancer cells *in vitro *[1,2].

Additionally, the results of these studies, along with those of our previous studies [1,2], were consistent with the hypothesis that the expression of p27 is regulated primarily at the level of translation. For more information about this issue, please go to the Methods section below,

Based on these results, we constructed a schematic diagram (Figure 1d) showing the outline of how 4-OH-tamoxifen could up-regulate the expression of p27, down-regulate the cell cycle progression from G1 to S phase, thereby inhibiting the DNA replication of the human breast cancer cells *in vitro*.

### Moderate increase in the concentration of D-(+)-glucose down-regulated the expression of p27, and, conversely, deficiency of D-(+)-glucose, L-leucine, L-methionine, L-cysteine or combination of L-methionine and L-cysteine up-regulated the expression of p27 in human MDA-MB-231 breast cancer cells in vitro

**Figure 2:**
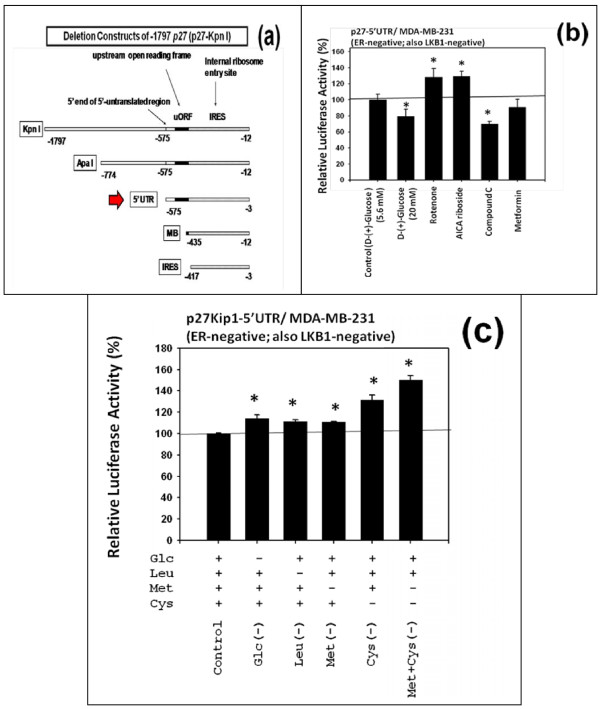
**Moderate increase in the concentration of D-(+)-glucose down-regulated, but deficiency of D-(+)-glucose, L-leucine, L-methionine, L-cysteine or combination of L-methionine and L-cysteine up-regulated the relative luciferase activity of p27-5'UTR in human MDA-MB-231 breast cancer cells in vitro**. (a) Schematic drawing of the luciferase reporter plasmid containing a proximal 5'-upstream region (-575) of the *p27 *gene (p27-5'UTR). (b) Moderate increase in the concentration of D-(+)-glucose down-regulated the relative luciferase activity of p27-5'UTR in MDA-MB-231 cells. This panel also shows that rotenone (inhibitor of NADH dehydrogenase (Complex 1) of the mitochondrial respiratory oxidation-phosphorylation chain) and AICA riboside (inhibitor of AMPK (5'-AMP-activated protein kinase)) up-regulated the relative luciferase activity of p27-5'UTR in MDA-MB-231 cells. In contrast, compound C (activator of AMPK) down-regulated the relative luciferase activity of p27-5'UTR in these cells. Metformin (the most widely prescribed anti-diabetic drug that activates AMPK in the cells by mechanisms that are dependent on its upstream kinase, the tumor suppressor LKB1) did not either up or down-regulate the relative luciferase activity of p27-5UTR probably because MDA-MB-231 cells lack LKB1. (c) This panel shows that deficiency of D-(+)-glucose, L-leucine, L-methionine, L-cysteine, or combination of L-methionine and L-cysteine up-regulated the relative luciferase activity of p27-5'UTR in MDA-MB-231 cells. All assays were performed in triplicates and repeated three times.

The effects of (a) moderate increase in the concentration of D-(+)-glucose and (b) deficiency of D-(+)-glucose, L-leucine, L-methionine, L-cysteine or combination of L-methionine and L-cysteine on the expression of p27 in MDA-MB-231 cells were investigated using one of the luciferase reporter plasmids containing a proximal 5'-upstream region of *p27 *gene (-575 p27-5'UTR) (Figure 2a).

The results indicated that moderate increase in the concentration of D-(+)-glucose down-regulated the relative luciferase activity of -575 p27-5'UTR in MDA-MB-231 cells (Figures 2b). In contrast, deficiency of D-(+)-glucose, L-leucine, L-methionine, L-cysteine or combination of L-methionine and L-cysteine up-regulated the relative luciferase activity of p27 in these cells (Figure 2c). It should be noted that deficiency of the combination of L-methionine and L-cysteine up-regulated the relative luciferase activity of p27 more than the deficiency of individual amino acids.

The results (Figure 2b) also indicated that (a) rotenone (inhibitor of NADH dehydrogenase (Complex 1) of the mitochondrial respiratory oxidation-phosphorylation chain) and AICA riboside (inhibitor of AMPK (5'-AMP-activated protein kinase)) up-regulated the relative luciferase activity of p27 in MDA-MB-231 cells, but (b) compound C (activator of AMPK) down-regulated the relative luciferase activity of p27 in these cells. Metformin did not either up or down-regulate the relative luciferase activity of p27 probably because MDA-MB-231 cells lack LKB1.

### Differential effects of 4-hydroxytamoxifen and deficiency of D-(+)-glucose on the upstream molecular signaling pathways of the expression of p27: pathways immediately downstream of mTORC1 (mammalian target of rapamycin complex 1)

**Figure 3:**
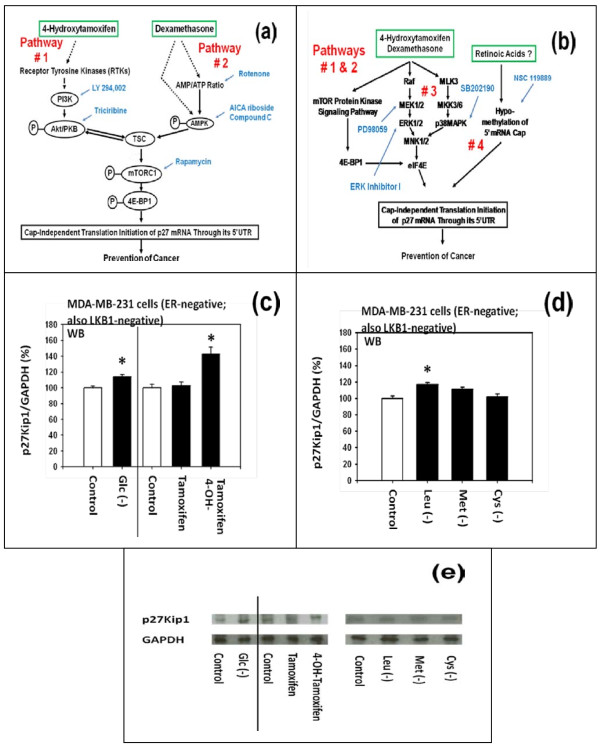
**Schematic diagram of the four different upstream molecular signaling pathways of p27 expression that could lead to either increased or decreased expression of p27 in human breast cancer cells in vitro**. (a) Previously, we identified and reported four different upstream molecular signaling pathways of the expression of p27 [1,2]. We also reported previously that 4-hydroxytamoxifen - but not tamoxifen - up-regulated the expression of p27 by using pathway #1 which consists mainly of receptor tyrosine kinases (RTKs) and mTORC1 [2]. Now, we hypothesize that (i) moderate increase in the concentration of D-(+)-glucose down-regulates and (ii) deficiency of D-(+)-glucose or certain L-amino acids up-regulates the expression of p27 using pathway #2 which consists mainly of AMPK and mTORC1. (b) We also identified and reported previously two additional upstream molecular signaling pathways - namely #3 and #4 - of the expression of p27 [1,2]. (c and e) Western immunoblot analysis of the effects of D-(+)-glucose deficiency, DMSO, tamoxifen and 4-hydroxytamoxifen on the expression of p27 protein in MDA-MB-231 cells. (d and e) Western immunoblot analysis of the effects of the deficiency of L-leucine, L-methionine or L-cysteine on the expression of p27 protein in these cells. All assays were performed in triplicates and repeated three times.

Previously, we identified and reported four different upstream molecular signaling pathways of p27 expression that could lead to either activation or inactivation of the translation initiation of p27 mRNA through its unusually long 5'-untranslated region (5'-UTR) (-575) of p27 mRNA (Figure 3a and 3b) [2]. We also reported previously that 4-hydroxytamoxifen (4-OH-tamoxifen) up-regulated the expression of p27 by using pathway #1 which consists mainly of receptor tyrosine kinases (RTKs) and mTORC1 (Figure 3a) [2]. We now hypothesize that (a) moderate increase in the concentration of D-(+)-glucose down-regulates and, conversely, (b) deficiency of D-(+)-glucose or certain L-amino acids up-regulates the expression of p27 by using pathway #2 which consists mainly of AMPK (5'-AMP-activated protein kinase) and mTORC1 (mammalian target of rapamycin complex 1) (Figure 3a).

To begin to test these hypotheses, we first performed the western immunoblot analysis of the expression of p27 protein itself. The results (Figures 3c, d and 3e) indicated that 4-OH-tamoxifen and deficiency of D-(+)-glucose or L-leucine up-regulated the expression of p27 protein, but deficiency of L-methionine or L-cysteine did not in MDA-MB-231 cells.

In order to look more closely into the effects of 4-OH-tamoxifen and deficiency of D-(+)-glucose or certain L-amino acids on the upstream molecular signaling pathways #1 and #2 of the expression of p27, western immunoblot analyses were performed to investigate the proteins immediately downstream of mTORC1, namely eukaryotic translation initiation factor 4E binding protein 1 (4E-BP1) and p70 S6 kinase 1 (S6K1).

#### (a) Differential effects on the phosphorylation of 4E-BP1

**Figure 4:**
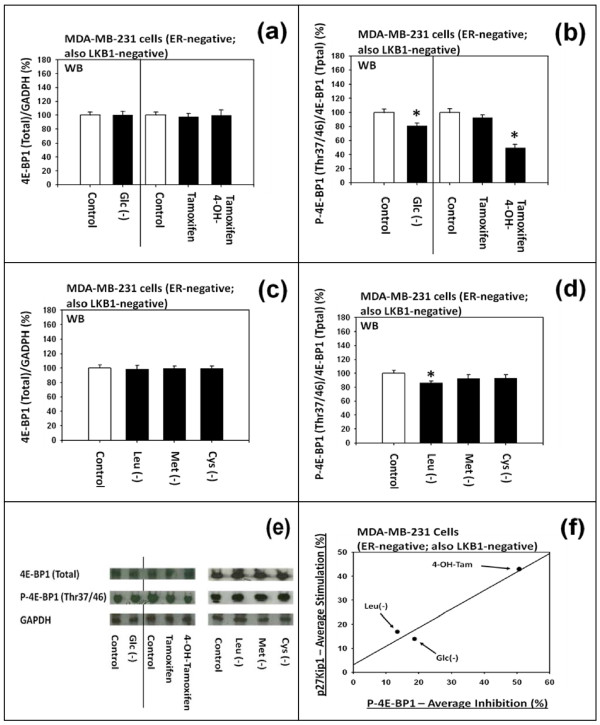
**Effects of tamoxifen, 4-hydroxytamoxifen, and deficiency of D-(+)-glucose, L-leucine, L-methionine or L-cysteine on the phosphorylation of 4E-BP1 in MDA-MB-231 cells**. (a and e) Western immunoblot analysis of the effects of D-(+)-glucose deficiency, DMSO, tamoxifen and 4-hydroxytamoxifen on the expression of total 4E-BP1 (eukaryotic translation initiation factor 4E binding protein 1) and (b and e) phosphorylated 4E-BP1. (c and e) Western immunoblot analysis of the effects of the deficiency of L-leucine, L-methionine, or L-cysteine on the expression of total 4E-BP1 and (d and e) phosphorylated 4E-BP1. All assays were performed in triplicates and repeated three times. (f) Correlation between the degree of expression of p27 protein and the degree of down-regulation of the phosphorylated 4E-BP1.

Figure 4a to 4e show that (a) 4-OH-tamoxifen and (b) deficiency of D-(+)-glucose or L-leucine did not either down or up-regulate the expression of total 4E-BP1, but they down-regulated the phosphorylated 4E-BP1. As summarized in Figure 4f, the degree of down-regulation of the phosphorylated 4E-BP1 appeared to be positively and linearly correlated with the degree of expression of p27.

#### (b) Differential effects on the phosphorylation of S6K1

**Figure 5:**
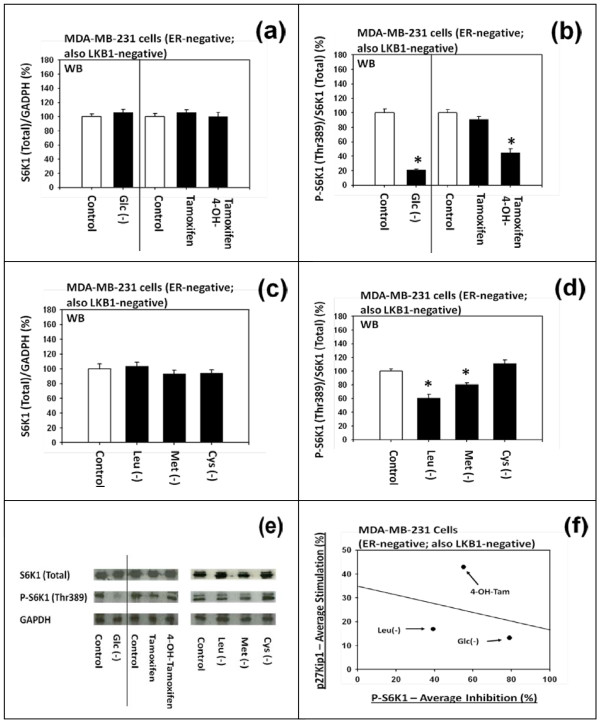
**Effects of tamoxifen, 4-hydroxytamoxifen, and deficiency of D-(+)-glucose, L-leucine, L-methionine or L-cysteine on the phosphorylation of S6K1 in MDA-MB-231 cells**. (a and e) Western immunoblot analysis of the effects of D-(+)-glucose deficiency, DMSO, tamoxifen and 4-hydroxytamoxifen on the expression of total S6K1 and (b and e) phosphorylated S6K1. (c and e) Westerm immunoblot analysis of the effects of the deficiency of L-leucine, L-methionine or L-cysteine on the expression of total S6K1 and (d and e) phosphorylated S6k1. All assays were performed in triplicates and repeated three times. (f) Correlation between the degree of expression of p27 protein and the degree of down-regulation of phosphorylated S6k1.

Figure 5a to 5e show that (a) 4-OH-tamoxifen and (b) deficiency of D-(+)-glucose, L-leucine or L-methionine did not influence the expression of total S6K1, but they down-regulated the phosphorylated S6K1. As summarized in the Figure 5f, the degree of down-regulation of the phosphorylated S6K1 did not appear to be correlated with the degree of expression of p27.

It should be noted that (a) 4-OH-tamoxifen and (b) deficiency of D-(+)-glucose or certain L-amino acids exerted differential effects on the degree of down-regulation of either the phosphorylated 4E-BP1 or phosphorylated S6K1. For example, (a) 4-OH-tamoxifen preferentially down-regulated the phosphorylation of 4E-BP1 over S6K1. Conversely, (b) D-(+)-glucose deficiency preferentially down-regulated the phosphorylation of S6K1 over 4E-BP1. (c) L-Leucine deficiency significantly down-regulated the phosphorylation of both 4E-BP1 and S6K1, but to a much lesser extent. (d) L-Methionine deficiency significantly down-regulated the phosphorylation of only S6K1 and to a much lesser extent; but it did not significantly down-regulate the phosphorylation of 4E-BP1. Lastly, (e) L-cysteine deficiency did not significantly down-regulate the phosphorylation of either 4E-BP1 or S6K1.

### Differential effects of 4-hydroxytamoxifen and deficiency of D-(+)-glucose on the upstream molecular signaling pathways of p27 expression: pathways further downstream of mTORC1

**Figure 6:**
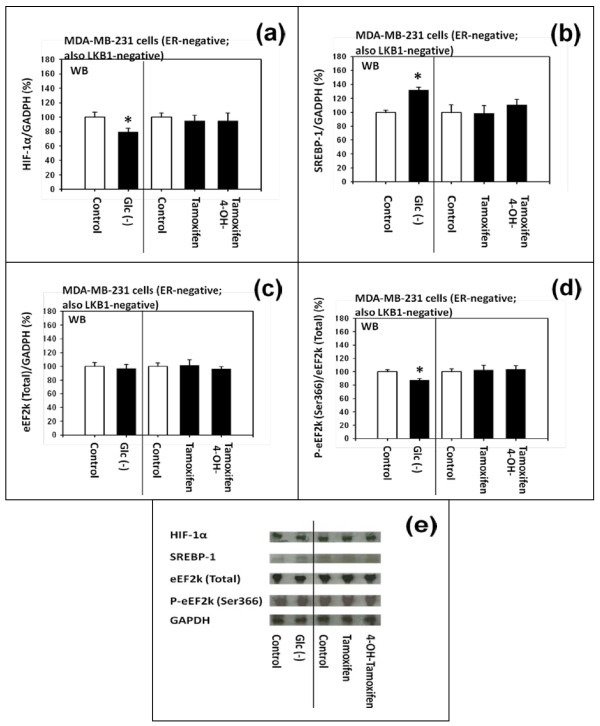
**Effects of tamoxifen, 4-hydroxytamoxifen and deficiency of D-(+)-glucose on the expression of HIF-1a, SREBP-1 and phosphorylation of eEF2k in MDA-MB-231 cells**. (a and e) Western immunoblot analysis of the effects of D-(+)-glucose deficiency, DMSO, tamoxifen and 4-hydroxytamoxifen on the expression of HIF1α. (b and e) SREBP-1, (c and e) total eEF2k and (d and e) phosphorylated eEF2k. All assays were performed in triplicates and repeated three times.

Next, we investigated the effects of tamoxifen, 4-OH-tamoxifen, and the deficiency of D-(+)-glucose on the pathways further downstream of mTORC1. They were (a) hypoxia-inducible factor 1α (HIF-1α), (b) sterol regulatory element binding protein-1 (SREBP-1) and (c) phosphorylation of eukaryotic elongation-factor-2 kinase (eEF2k). The results of the western immunoblot analyses are presented in Figure 6a to 6e. The effects of L-amino acid deficiencies were not investigated because they exerted either only a moderate or no effect on the phosphorylation of 4E-BP1 or S6K1.

#### (a) Differential effects on HIF-1α

HIF-1α has been variably characterized in the literature as being a protein downstream of 4E-BP1, S6K1 or both. The results of our western immunoblot analyses presented in Figure 6a and 6e indicated that D-(+)-glucose deficiency significantly down-regulated the expression of HIF-1α; but 4-OH-tamoxifen did not. These results are consistent with the hypothesis that HIF-1α is a protein primarily downstream of S6K1.

#### (b) Differential effects on SREBP-1 and phosphorylated eEF2k

No controversy exists in the literature as to the SREBP1 and eEF2k; the consensus is that they are the proteins primarily downstream of S6K1. The results of our western immunoblot analyses of SREBP1 (Figure 6b and 6e) and phosphorylated eEF2k at Ser366 (Figure 6c, d and 6e) are consistent with this consensus.

### Differential effects of 4-hydroxytamoxifen and deficiency of D-(+)-glucose or L-leucine on the upstream molecular signaling pathways of p27 expression: pathways upstream of mTORC1

The results presented above (Figure 2b) suggested that NADH dehydrogenase (Complex 1) in the mitochondrial respiratory oxidation-phosphorylation chain and 5'-AMP-activated protein kinase (AMPK) are the two critical components of the pathway #2 upstream of mTORC1. In addition to these two proteins, we investigated two other proteins that also appeared to be associated with the pathway #2 upstream of mTORC1. They were mitochondrial ATP Synthase α chain (ATP5A) in the Complex V of respiratory oxidation-phosphorylation chain and mitochondrial SIRT3.

#### (a) Differential effects on the mitochondrial ATP5A

During our preliminary proteomic analysis of the hepatic proteins of genetically obese mice and long-lived dwarf mice, we observed that mitochondrial ATP5A was most significantly down-regulated in the liver of leptin-deficient obese mice relative to the lean control mice. Conversely, we also observed that mitochondrial ATP5A was most significantly up-regulated in the liver of long-lived Ames dwarf mice compared to the normal Ames mice. Based on these preliminary observations, we decided to investigate the effects of 4-OH-tamoxifen and deficiency of D-(+)-glucose or certain L-amino acids on the expression of mitochondrial ATP5A in the human MDA-MB-231 breast cancer cells *in vitro*.

**Figure 7:**
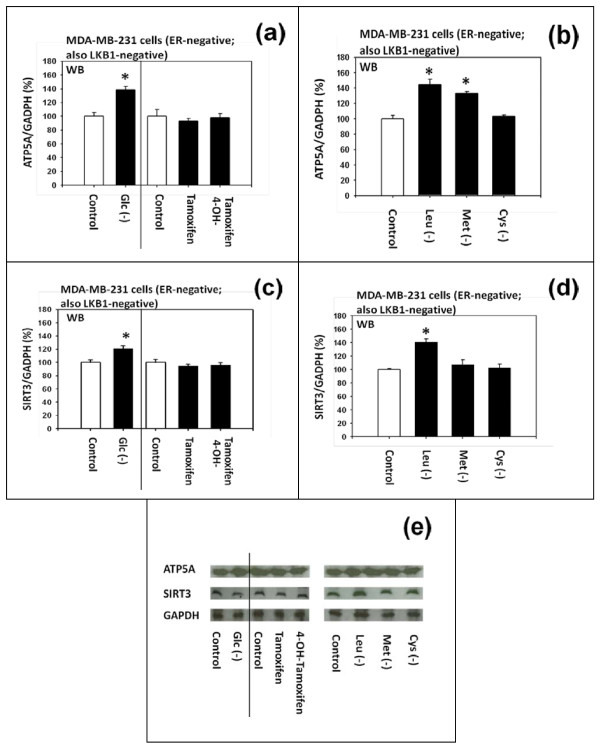
**Effects of tamoxifen, 4-hydroxytamoxifen and deficiency of D-(+)-glucose, L-leucine, L-methionine or L-cysteine on the expression of mitochondrial ATP5A and SIRT3 in MDA-MB-231 cells**. (a and e) Western immunoblot analysis of the effects of D-(+)-glucose deficiency, DMSO, tamoxifen, 4-hydroxytamoxifen and (b and e) deficiency of L-leucine, L-methionine or L-cysteine on the expression of mitochondrial ATP5a in the Complex V of respiratory oxidation-phosphorylation system. (c and e) Western immunoblot analysis of the effects of D-(+)-glucose deficiency, DMSO, tamoxifen, 4-hydroxytamoxifen and (d and e) deficiency of L-leucine, L-methionine or L-cysteine on the expression of mitochondrial SIRT3. The SIRT3 is one of the seven mammalian anti-aging and anti-metabolic sirtuins. All assays were performed in triplicates and repeated three times.

The results of our western immunoblot analyses (Figure 7a, b and 7e) indicated that 4-OH-tamoxifen did not influence the expression of mitochondrial ATP5A, but deficiency of D-(+)-glucose, L-leucine or L-methionine up-regulated it. Deficiency of L-cysteine did not alter the expression of mitochondrial ATP5A.

#### (b) Differential effects on the mitochondrial SIRT3

Mitochondrial SIRT3 is one of the seven mammalian anti-aging and anti-metabolic sirtuins. It was reported recently that mitochondrial ATP5A forms complex with and interacts with mitochondrial SIRT3 [24]. Based on this report, we decided to investigate the effects of 4-OH-tamoxifen and deficiency of D-(+)-glucose or certain L-amino acids on the expression of mitochondrial SIRT3 in the human MDA-MB-231breast cancer cells *in vitro*.

The results of our western immunoblot analyses (Figure 7c, d and 7e) indicated that deficiency of D-(+)-glucose or L-leucine - but not 4-OH-tamoxifen - up-regulated the expression of mitochondrial SIRT3 in these cells. Deficiency of L-methionine or L-cysteine, however, did not either up or down-regulate the expression of SIRT3.

Finally, 4-OH-tamoxifen and deficiency of D-(+)-glucose or certain L-amino acids did not regulate the expression of nuclear anti-aging and anti-metabolic protein SIRT1 in these cells (results not shown).

## Discussion

**Figure 8:**
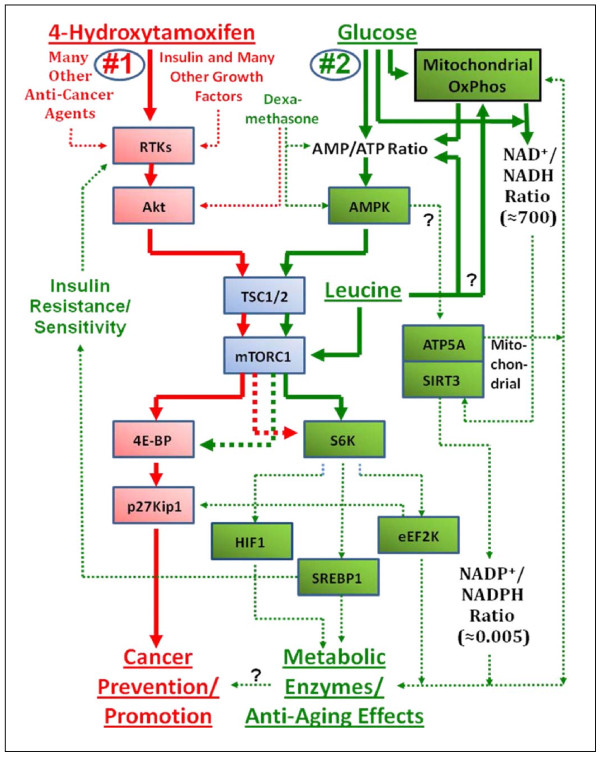
**Schematic diagram of the hypothetical molecular signaling pathways of the expression of p27 by which moderate increase in the concentration of D-(+)-glucose down-regulates and deficiency of D-(+)-glucose or L-leucine up-regulates the expression of p27**. This summary diagram shows the outline of how 4-hydroxytamoxifen uses primarily pathway #1 to up-regulate the expression of p27, arrest the G1-to-S phase transition of cell cycle, and inhibit DNA replication in human breast cancer cells *in vitro*. 4-Hydroxytamoxifen preferentially phosphorylates 4E-BP1 over S6K1. The diagram also shows the outline of how the deficiency of D-(+)-glucose uses primarily pathway #2 to up-regulate the expression of p27, arrest the G1-to-S phase transition of cell cycle, and inhibit DNA replication in human breast cancer cells *in vitro*. D-(+)-Glucose deficiency preferentially phosphorylates S6K1 over 4E-BP1. The diagram also shows that the deficiency of L-leucine enters the pathway #2 at points different from the deficiency of D-(+)-glucose. Finally, the diagram shows that deficiency of D-(+)-glucose or L-leucine uses L-upstream molecular signaling pathway #2 of the expression of p27 to up-regulate the phosphorylation of AMPK and the expression of mitochondrial ATP5A and SIRT3. The mitochondrial SIRT3 exerts anti-aging and other metabolic effects on the cells.

Based on the results presented above, a schematic diagram is presented in Figure 8 that outlines the effects of 4-hydroxitamoxifen, moderate increase in the concentration of D-(+)-glucose and deficiency of D-(+)-glucose or L-leucine on the pathways #1 and #2 of the upstream molecular signaling pathways of the expression of p27 in human breast cancer cells *in vitro*. The results presented are consistent with the following hypotheses, namely:

(a) 4-Hydroxitamoxifen up-regulates the expression p27 in human breast cancer cells in vitro primarily by using pathway #1. The pathway #1 consists mainly of receptor tyrosine kinases/phosphoinositide-3-kinase/Akt/tuberous sclerosis complex/mammalian target of rapamycin complex 1/eukaryotic translation initiation factor 4E-binding protein 1 (RTKs/PI3K/Akt/TSC/mTORC1/4E-BP1).

(b) Moderate increase in the concentration of D-(+)-glucose or certain amino acids down-regulates and deficiency of D-(+)-glucose or L-leucine up-regulates the expression of p27 in human breast cancer cells in vitro primarily by using pathway #2. The pathway #2 consists mainly of 5'-AMP-activated protein kinase/tuberous sclerosis complex/mammalian target of rapamycin complex 1/p70 S6 kinase I (AMPK/TSC/mTORC1/S6K1). The pathway #2 also modulates the phosphorylation of 4E-BP1 thereby regulating the expression of p27, but this effect is secondary to its effect on the phosphorylation of S6K1.

Down-regulation of the phosphorylation of S6K1 in the pathway #2 by the deficiency of D-(+)-glucose resulted in the (i) down-regulation (de-stabilization) of HIF-1α, (ii) up-regulation (stabilization) of SREBP1 and (iii) down-regulation of the phosphorylation of eEF2k.

The SREBP1 is of particular interest here because SREBP1 has recently been implicated in the insulin resistance of type 2 diabetes. It is well known that, in the liver of many insulin-resistant mouse models, insulin fails to suppress D-(+)-glucose production (gluconeogenesis) but continue to promote lipid synthesis. It is also known that mTORC1 down-regulates (activates or de-stabilizes) SREBP-1 and thereby uncouples lipogenesis from gluconeogenesis [25]. This down-regulation of the expression of SREBP1 by mTORC1 appears to be achieved at least in part by promoting its posttranslational processing via S6K1, which in turn leads to the increased transcription of genes involved in sterol and lipid biosynthesis and the oxidative arm of the pentose-phosphate pathway [26,27]. mTORC1-mediated decrease in (or activation or de-stabilization of) the expression of SREBP-1 also appears to be necessary for lipogenesis induced by Akt [28]. In summary, our results suggested that the deficiency of D-(+)-glucose could suppress insulin resistance and restore insulin sensitivity by down-regulating the phosphorylation of S6K1 and up-regulating (de-activating or stabilizing) the expression of SREBP1,

### Deficiency of D-(+)-glucose or L-leucine - but not 4-hydroxitamoxifen - up-regulates the expression of mitochondrial ATP Synthase α chain (ATP5A) in the Complex V of respiratory oxidation-phosphorylation chain

During our preliminary proteomic analysis of the hepatic proteins of leptin-deficient obese mice and long-lived dwarf mice, we observed that the expression of mitochondrial ATP5A protein was most significantly down-regulated in the liver of leptin-deficient obese mice relative to the lean control mice. We also observed that ATP5A protein was most significantly up-regulated in the liver of long-lived Ames dwarf mice relative to the normal Ames mice.

Based on these findings, we decided to investigate and, in fact, reported, as described above in the results section, that the deficiency of D-(+)-glucose, L-leucine or L-methionine up-regulated the expression of mitochondrial ATP5A in the human MDA-MB-231 breast cancer cells *in vitro *as well.

As to the possible molecular mechanisms of the up-regulation of ATP5A, we realized that almost no study was published in the literature. One exception was the study published in 2010, where authors speculated that, as wakefulness continues in mice, the maintenance of ATP becomes more challenging and likely to involve additional nuclear transcriptional mechanisms [29]. The authors further stated that, initially, the demand for increased ATP during wakefulness is met by increased activity of the mitochondrial respiratory oxidation-phosphorylation (OxPhos) system (including ATP5B and probably ATP5A as well). This would eventually lead to an increase in the production of reactive oxygen species (ROS) during extended wakefulness that would then lead to uncoupling with at least temporary decline in ATP and increase in AMP resulting in the activation (increased phosphorylation) of 5'-AMP-dependent protein kinase (AMPK).

This study [29] places the molecular basis of the metabolic up-regulation of the expression of ATP5A by the deficiency of D-(+)-glucose or L-leucine at the AMPK, which is one of the essential components of the pathway #2 in the upstream molecular signaling pathways of p27 expression.

### Deficiency of D-(+)-glucose or L-leucine - but not 4-hydroxitamoxifen - up-regulates the expression of mitochondrial SIRT3, one of the seven mammalian anti-aging and anti-metabolic sirtuins

Mitochondrial SIRT3 is one of the seven mammalian sirtuins that are involved in anti-aging and other metabolic processes. Recently, it was reported that mitochondrial SIRT3 forms complex with and interacts with mitochondrial ATP5A [24]. Since SIRT3 is known to be present ubiquitously in the body, we speculated that SIRT3 could also be present in the human MDA-MB-231 breast cancer cells *in vitro *and, in fact, as described in the results section above, *w*e found that deficiency of D-(+)-glucose or L-leucine - but not 4-hydroxytamoxifen - up-regulated the expression of SIRT3 in these cells.

Sirtuins are a family of NAD^+^-dependent protein deacetylases that regulate cellular functions through deacetylation of a wide range of protein targets [24,30-34]. Overexpression of *Sir2*, the first gene discovered in this family, is able to extend the life span in various organisms. The anti-aging effects of human homologues of sirtuins, SIRT1-7, have also been suggested by animal and human studies.

The results of our study are consistent with the notion that deficiency of D-(+)-glucose or L-leucine - but not 4-hydroxytamoxifen - could exert anti-aging and other metabolic effects through the pathway that involves AMPK, ATP5A and SIRT3. It has been reported, in fact, that the expression of mitochondrial SIRT3 may be up-regulated in caloric restriction and down-regulated in obesity and diabetes [35,36]. It has also been reported that the activation (increased phosphorylation) of AMPK may lead to up-regulation of the expression of mitochondrial SIRT3 [37].

Thus, the origin of the metabolic up-regulation of the expression of mitochondrial SIRT3 by the deficiency of D-(+)-glucose or L-leucine could be traced to AMPK, which is again one of the essential components of the pathway #2 in the upstream molecular signaling pathways of p27. expression.

## Conclusions

Previously, we identified and reported four different upstream molecular signaling pathways - we called them pathway #1, #2, #3 and #4 - of the expression of p27 in human breast cancer cells in vitro. Based on the results presented above, we conclude that:

(a) 4-Hydroxitamoxifen uses primarily pathway #1 to up-regulate the expression of p27. The pathway #1 consists mainly of receptor tyrosine kinases (RTKs) and mammalian target of rapamycin complex 1 (mTORC1).

(b) Moderate increase in the concentration of D-(+)-glucose is likely to use primarily pathway #2 to down-regulate the expression of p27. The pathway #2 consists mainly of 5'-AMP-activated protein kinase (AMPK) and mTORC1 protein kinase.

(c) Deficiency of D-(+)-glucose or L-leucine uses primarily pathway #2 to up-regulate the expression of p27.

(d) Additionally, deficiency of D-(+)-glucose or L-leucine - but not 4-hydroxytamoxifen - also up-regulates the expression of mitochondrial ATP5A in the Complex V of respiratory oxidation-phosphorylation chain and mitochondrial anti-aging as well as anti-metabolic SIRT3.

## Methods

### Reagents

4-Hydroxytamoxifen, tamoxifen, D-(+)-glucose, and rotenone were obtained from Sigma-Aldrich (St. Louis, MO, USA). Compound C and metformin were obtained from Calbiochem/EMD (San Diego, CA, USA). AICA riboside was purchased from Phoenix Pharmaceuticals, Inc. (Belmont, CA, USA). Dulbecco's Modified Eagle's Medium (DMEM) Labeling Kit was purchased initially from Chemicon International (Temecula, CA, USA) and later from EMD Millipore (Billerica, MA, USA).

The following antibodies were purchased from Cell Signaling Technology, Inc. (Danvers, MA, USA): namely (a) total 4E-BP1 and phospho-4E-BP1 (Thr37/46); (b) total S6K1 and phospho-S6K1 (Thr389); and (c) total eEF2k and phospho-eEF2k (Ser366). Additionally, the following antibodies were purchased from Santa Cruz Biotechnology, Inc. (Santa Cruz, CA, USA): namely (a) p27, (b) GAPDH, (c) ATP5A, (d) SIRT3, (e) SIRT1, (f) SREBP-1, and (g) HIF-1α.

### Cell Cultures

Human MDA-MB-231 breast cancer cells (estrogen receptor (ER) and LKB1-double negative) were purchased from the American Type Culture Collection (Rockville, MD, USA). The cells were grown in Dulbecco's Modified Eagle's Medium (DMEM) containing 4.5 g/L of D-(+)-glucose, supplemented with 10% heat-inactivated FBS, 2% L-glutamine, and antibiotics/antimycotics. Incubation of the cells was carried out at 37°C in a 5% CO_2 _humidified chamber. The cells were subcultured after trypsinization with 0.05% trypsin-0.02% EDTA solution. The cells were always maintained below confluency and checked periodically for mycoplasmal infection by DNA fluorochrome staining.

### Plasmids

Luciferase reporter plasmids containing one of the following proximal 5'-upstream regions of the *p27 *gene were used to transfect the human MDA-MB-231 breast cancer cells: -1797 *p27 *(p27-Kpn I) [38], -774 *p27 *(p27-Apa I) [38], and -575 *p27 *(p27-5'UTR) [4,8]. The control luciferase reporter plasmids not containing these inserts were also prepared and used to test if 24-hour treatment of the cells with DMSO, 4-hydroxytamoxifen, tamoxifen, excess D-(+)-glucose, or the deficiency of D-(+)-glucose, L-leucine, L-methionine, L-cysteine, or combination of L-methionine and L-cysteine was exerting any spurious effects on the backbone, rather than the insert, of the luciferase reporter plasmids. None of these treatments were found to exert any spurious effects on the backbone of the plasmids in the human MDA-MB-231 breast cancer cells.

### Transfection and Luciferase Assay

Transfections were performed according to the published protocol [39] using FuGENE 6 obtained from Roche Applied Science (Indianapolis, IN, USA). In brief, 24 hours before reporter transfection, the cells were seeded into a 60-mm tissue culture dish at a density of 1.5 × 10^5 ^cells/dish and incubated at 37°C in a 5% CO2 humidified chamber. Transfection of the luciferase reporter plasmid was then carried out with 1 μg of luciferase reporter plasmid and 0.2 μg of pSV-β-galactosidase internal control plasmid (Promega, Madison, WI, USA) mixed with 3 μL of FuGENE 6 solution in 3 mL of FBS-free DMEM supplemented with only 2% L-glutamine. A minimum of 5-hour incubation at 37°C was needed for transient transfection, followed by 18-hour incubation in DMEM with 10% FBS for recovery. The transfected cells were then starved in DMEM with 0.2% FBS for 24 hours. Subsequently, the resulting cells were cultured either (a) in the presence of DMSO, tamoxifen, or 4-hydroxytamoxifen in the regular DMEM with 0.2% FBS, (b) in the presence of a moderate increase in the concentration of D-(+)-glucose or (c) deficiency of D-(+)-glucose, L-leucine, L-methionine, L-cysteine or combination of L-methionine and L-cysteine in the appropriately supplemented basal DMEM Labeling Kit as described in the figure legends. After 24 hours, the treated cells were collected and lysed using Reporter Lysis Buffer (Promega, Madison, WI) and the resulting cell lysates were assayed for luciferase activity using Luciferase Assay Kit (Promega, Madison, WI, USA) and TD-20/20 Luminometer (Turner Designs, Sunnyvale, CA, USA). β-Galactosidase activity was measured using chlorophenol red-β-D-galactopyranoside (CPRG) (Sigma-Aldrich, St. Louis, MO, USA) as a substrate.

Each luciferase activity driven by a specific proximal 5'-upstream region of the *p27 *gene was normalized to β-galactosidase activity in order to control for variations in transfection efficiency.

As for the issue of whether the expression of p27 was regulated primarily at the level of translation, we performed the following three different studies:

(a) The various deletion constructs of -1797 p27-Kpn1 luciferase reporter plasmids were used to determine the core element of the activation of the proximal 5'-upstream region (-1797) of *p27 *gene [1,2]. The results indicated that various nutritional and chemopreventive anti-cancer agents, including tamoxifen and 4-hydroxytamoxifen, activated the proximal 5'-upstream region (-1797) of p27 gene through its 5'-untranslated region (5'UTR) (-575). It is well established that this region mediates the cap-independent translation initiation of p27 mRNA [4-10].

(b) To investigate if -575 p27 (p27-5'UTR) contains any cryptic transcription factor binding sites - in other words, if the expression of p27 is regulated primarily at the level of transcription - the luciferase activity of the region was stimulated with 4-hydroxytamoxifen in the presence of an adequate dose of antibiotic actinomycin D [1,2]. Actinomycin D is a well-known inhibitor of transcription. The results indicated that the -575 p27 (5'-untranslated region (5'UTR)) is unlikely to contain any cryptic transcription factor binding sites. This assay was performed not only with tamoxifen and 4-hydroxytamoxifen, but also with many other anti-cancer agents.

(c) Depending on the cell types, it was observed from time to time that control vector expression was affected by each treatment and also there could be cell cycle effects probably changing with treatment. To exclude these possibilities, the p27 luciferase reporter vector that does not contain and insert of the specific proximal 5'-upstream region of the *p27 *gene was prepared and tested using the same anti-cancer agents and cell types [1,2]. In these exceptional cases, the following formula was used to correct this false increase in the relative luciferase activity:

Relative luciferase activity (% )=(Experimental luciferase activity/Control luciferase activity)×100,

where,

(1) Experimental luciferase activity = {Test compound/None}{Luciferase reporter vector containing a specific insert},

(2) Control luciferase activity = {Test compound/None}{Luciferase reporter vector NOT containing a specific promoter insert}, and

(3) Test compound/None = [Luc(Test)/βGal(Test)]/[Luc(None)/βGal(None)].

The human MDA-MB-231breast cancer cells that were used in this study did not present any of these exceptional problems. For additional information on this and related issues, please refer to the reference #40 [40].

### Western Immunoblot Analysis

Western immunoblot analysis of the upstream molecular signaling pathways of the expression of p27 was performed using estrogen receptor (ER) and LKB1-double negative human MDA-MB-231 breast cancer cells *in vitro*. This analysis was performed without either transfecting the cells with various proximal 5'-upstream region of *p27 *gene-luciferase reporter plasmids or adding any growth factors to avoid the artificial stimulation of the cell proliferation.

In brief, the cells were seeded at a density of 5.5 × 10^6 ^cells/dish into a 100-mm tissue culture dish containing 10 mL of DMEM supplemented with 10% heat-inactivated fetal bovine serum (FBS), 2% L-glutamine, and antibiotic/antimycotic solution and incubated at 37°C in a 5% CO_2 _humidified chamber for 24 hours. After 24 hours, the cells were partially synchronized for another 24 hours in DMEM containing only 0.2% (v/v) of FBS. Subsequently, the resulting cells were cultured either (a) in the presence of DMSO, tamoxifen, or 4-hydroxytamoxifen in the regular DMEM with 0.2% FBS or (b) in the presence or absence of D-(+)-glucose, L-leucine, L-methionine, or L-cysteine in the appropriately supplemented basal DMEM Labeling Kit as described in the figure legends. After 24 hours, the cells were washed twice with cold 1× PBS and scraped in 1× RIPA Lysis Buffer (Santa Cruz Biotechnology, Santa Cruz, CA, USA) containing phenylmethylsulphonyl fluoride (PMSF), protease inhibitor cocktail, sodium orthovanadate and 50 mM NaF. The cells were then sonicated and the supernatant was collected by centrifugation and stored at -80°C.

The supernatants (60 μg protein/lane) were applied to the SDS-PAGE and, after fractionation, proteins were transferred to nitrocellulose membrane, which was then blocked and incubated in a solution containing first primary antibody. After shaking overnight at 4°C, the target proteins bound to the first primary antibody were further incubated in a solution containing alkaline phosphatase (AP)-conjugated secondary anti-immunoglobulin antibody and detected by chemiluminescence using TROPIX Western-Star Kit (Applied Biosystems, Foster City, CA, U.S.A.). After exposure to X-ray film, the blots were stripped using Western Re-Probe Solution (G-Biosciences, St. Louis, MO, U.S.A.), checked for removal of the chemiluminescence and then re-probed with second primary antibody.

Densitometric measurement of the intensity of the bands on the X-ray film was performed using UN-SCAN-IT Gel & Graph Digitizing Software Version 6.1 (Silk Scientific Corporation, Orem, UT, U.S.A.). Background corrections were done by four corner interpolation and optical density calculations were performed

### Statistical Analysis

An experimental value with statistical significance of P ≤ 0.05 compared to the control by t test is indicated as a single asterisk on top of the vertical bar.

## List of abbreviations

**Nonstandard abbreviations**: p27: p27Kip1; p21: p21Cip1/Waf1; AMPK: 5'-AMP-activated protein kinase; TSC: tuberous sclerosis complex; mTORC1: mammalian target of rapamycin complex 1; RTK: receptor tyrosine kinase; PI3K: phosphoinositide 3-kinase; PKB: protein kinase B; AMPK: 5'-AMP-activated protein kinase; MAPK: mitogen-activated protein kinase; MEK: mitogen-activated protein (MAP) kinase kinase; ERK: ERK MAP kinase; MNK: MAP kinase interacting kinase; m^7^G: 7-methylguanosine; CDK: cyclin-dependent kinase; CDI: cyclin-dependent kinase inhibitor; MNU: *N*-methyl-*N*-nitrosourea; ER: estrogen-receptor; 5'-UTR: 5'-untranslated region; IRES: internal ribosome entry site; DMSO: dimethyl sulfoxide; pGL3: pGL3 luciferase reporter vector; AdoMet or SAM, S-(5'-adenosyl)-L-methionine; AdoHcy or SAH: S-(5'-adenosyl)-L-homocysteine; 4E-BP1: eukaryotic translation initiation factor 4E binding protein 1; S6K: p70 S6 kinase; AICAR: 5-amino-4-imidazolecarboxamide aminoimidazole carboxamide ribonucleotide; Glc: D-(+)-glucose; Ser: L-serine; Thr: L-threonine; Met: L-methionine; Cys: L-cysteine; Leu: L-leucine; Tyr: L-tyrosine; eIF4E: eukaryotic translation initiation factor 4E; uORF: 5'-upstream open reading frame; FBS: fetal bovine serum; DMEM: Dulbecco's modified Eagle's medium; EDTA: ethylenediaminetetraacetic acid; CPRG: chlorophenol red-β-D-galactopyranoside; βGal: β-galactosidase; Luc: firefly luciferase; GAPDH: glyceraldehydes phosphate dehydrogenase; mTORC1: mammalian target of rapamycin complex 1; HIF-1α: hypoxia-inducible factor 1α; SREBP1: sterol regulatory element binding protein-1; eEF2k: eukaryotic elongation-factor-2 kinase; ATP5A: mitochondrial ATP Synthase α chain in the Complex V of respiratory oxidation-phosphorylation chain; SIRT3: sirtuin 3; SIRT1: sirtuin 1; OxPhos: mitochondrial oxidation-phosphorylation (repiratory electron transfer) chain, ROS, reactive oxygen species.

## Competing interests

The author declares that they have no competing interests.
